# Planetary health and health education in Brazil: Facing inequalities

**DOI:** 10.1016/j.onehlt.2022.100461

**Published:** 2022-11-17

**Authors:** Walter Leal Filho, José Baltazar Salgueirinho Osório de Andrade Guerra, Ana Regina de Aguiar Dutra, Maria Gabriela Mendonça Peixoto, Jefferson Traebert, Gustavo J. Nagy

**Affiliations:** a Manchester Metropolitan University, Department of Natural Sciences, Chester Street, Manchester M1 5GD, UK; b University of Southern Santa Catarina (UNISUL), Centre for Sustainable Development/Research Group on Energy Efficiency and Sustainability (GREENS), Florianopolis, Santa Catarina, Brazil; c Cambridge Centre for Environment, Energy and Natural Resource Governance, (CEENRG), University of Cambridge, Cambridge, UK; d Federal University of Viçosa - Rio Paranaíba Campus. Rio Paranaíba, Minas gerais, Brazil; e Graduate Program in Health Sciences, University of Southern Santa Catarina, Florianopolis, Santa Catarina, Brazil; f Instituto de Ecología y Ciencias Ambientales y Ecología, Facultad de Ciencias, Universidad de la República, Uruguay; g Hamburg University of Applied Sciences, Faculty of Life Sciences, Hamburg, Germany

**Keywords:** Planetary health, Education, Sustainable development goals, Inequalities, Brazil

## Abstract

Brazil has the world's fifth-largest population and seventh-largest economy. However, it also has many inequalities, especially in health education, which impacts health sector services. Thus, this article aims to describe the situation of planetary health and health education in Brazil, identifying how current policies support the cause of planetary health. This study had a qualitative approach characterised as exploratory research based on an integrative review and documentary research. The results show that, in recent decades, there have been positive improvements to achieve collective and planetary health, which advocates empathy and pro-environmental and humanitarian attitudes. However, the pursuit of planetary health in Brazil is being influenced by various challenges, ranging from the need for a sound policy framework to provisions of education and training on planetary health. Based on the need to address these deficiencies, the paper suggests some measures which should be considered as part of efforts to realise the potential of planetary health in the fifth largest country in the world.

## 1 Introduction

Whitmee et al. (2015), in the context of the report The Rockefeller Foundation-Lancet, call attention to guaranteeing planetary health. Whitmee and colleagues [1] pointed out that discussions on planetary health help to develop guidelines for the integration of human health, civilisation and environmental degradation, including the implementation of transforming actions and changes in governance systems to integrate social, economic and environmental policies, use interdisciplinary knowledge, and further the adoption of solutions that correct inequalities [1,2].

The authors mentioned in the previous paragraph call our attention to the use of interdisciplinary knowledge presented by a group of professionals from different areas of activity to deal with the planetary health agenda, which was also pointed out by the study of Sássi et al. [3]. In addition, Pettan-Brewer et al. [4] enhance Sássi et al.'s discussion when they state that it is necessary to encourage collaborative partnerships among different players, mainly from communities, to promote the health not only of people but also of animals, plants, the environment and the entire planet, with consequent significant results for planetary health by developing a healthier world with inclusion, equity and equality. Finally, from the perspective of inequality, Xu et al. [5] concluded that populations of less-developed Brazilian cities with socioeconomic inequalities might suffer more from temperature variations.

Planetary health emerged in the last decade as a new field of research, emphasising the impacts of human activities on the planet's health [6,7], as opposed to the impacts of natural events. For example, Ebi et al. [8] have extensively discussed the impacts of human activities on the health of people, fauna and flora, oceans, soil and the elements of the planet and also contributed with guidelines to mitigate these impacts.

In this scenario, health education plays a fundamental role. However, it was only from the 2000s onwards that health education acquired the character of State Policy in Brazil [9]. Among the different challenges and opportunities, it can be highlighted the establishment of initiatives related to the reorientation of professional training, with the permanent education of health workers, with emphasis on the integration between higher education institutions, health services and the community. There are many advances; however, there are challenges to be overcome and proposals to be implemented that collaborate to improve the problem-solving capacity of the Brazilian National Public Health System (*Sistema Único de Saúde* – SUS). Among the different inducing strategies, the technical, political and financial investments for management qualification significantly impact conducting public health policies. The enormous challenge of training and qualifying workers for the SUS in a way that aligns with the health needs of the population implies channeling efforts towards the construction and reconstruction of educational processes in the field of health education, understanding them as a substantial part of a strategy of institutional change, and not just to reach a specific objective.

Giulio et al. [10] point out that knowledge, teaching, practice, and research on health issues go beyond regional and national geographic boundaries and their social and environmental determinants. Thus, solutions require interventions and agreements between different actors, including countries, governments, and public and private institutions. Likewise, and in this context, Planetary Health assumes that health problems and the definition of public policies to address them cannot be separated from the current ecological emergency [11]. Planetary health presupposes a broader and deeper awareness of the interconnected problems that affect global health and their complex causes. It requires a paradigm shift in how the health-disease process is conceived, with the need for a deepening of syndemic knowledge and acceptance of the interconnection of health problems, environmental problems, socioeconomic dynamics, and how this whole scenario impacts population groups and communities in different ways, especially those with greater social vulnerability in a country with deep social inequalities such as Brazil.

Therefore, this article relies on the following research question: what is the role of current policies in the planetary health and health education in Brazil scenarios under the sustainable development agenda perspective?

The article is structured as follows. 2, 3 introduce the Health Sector and the trends in Health Education in Brazil, respectively. Then Section 4 explains the methodology, and Section 5 presents and discusses the results. Finally, Section 6 concludes the article.

## 2 The health sector in Brazil

According to Guzmán et al. [12], a cohesive framework to guide planetary health education institutions, educators, and learners does not exist. A task force of thought leaders in planetary health and education was convened by the Planetary Health Alliance to create the Planetary Health Education Framework and address this gap. They have considered five foundational domains that are believed to comprise the essence of planetary health knowledge, values, and practice. These domains are interconnection within nature, the Anthropocene and health, systems thinking and complexity, equity and justice, movement building, and systems change.

Public policies at any level of governance must jointly incorporate these domains, as they are inseparable. However, the second domain focuses on understanding how specific anthropogenic impacts on Earth's natural systems are connected to health outcomes. Understanding the links between the Anthropocene and health requires a social and ecological approach to health promotion and disease prevention and control, ranging from individual to population determinants of human, animal, and ecosystem health.

Health, as a complex social phenomenon, is dependent on the social structure and is determined by conditions that can be grouped under a tripod of sustainable development, such as economic development (economic growth, combating poverty, reduction of social inequalities), social development (demography, economy, and income, women's empowerment, education, governance, nutrition, health system structure) and environmental protection (geography, basic sanitation, safe and sustainable energy sources). The determinants alone are themes of several SDGs. For example,

SDG 1 - End poverty in all its forms, everywhere.

SDG 2 - End hunger, achieve food security and improved nutrition, and promote sustainable agriculture.

SDG 4 - Ensure inclusive and equitable quality education, and promote lifelong learning opportunities.

SDG 6 - Ensure the availability and sustainable management of water and sanitation.

SDG 7 - Ensure reliable, sustainable, modern, and affordable access to energy.

SDG 8 - Promote sustained, inclusive, and sustainable economic growth, full and productive employment, and decent work.

Nevertheless, SGD 3 implies ensuring healthy lives and promoting well-being for all. This goal was translated into Brazil as ensuring, through the SUS, universal health coverage, access to essential quality health services at all levels of care, and access to safe, effective, and quality essential medicines and vaccines incorporated into the products offered by SUS. The wording of the global goal was adapted to the text of the Brazilian Constitution in its article 194, which determines that social security comprises an integrated set of actions initiated by public authorities and society aimed at ensuring health, welfare, and social assistance. Thus, considering the universal character of the SUS, it became more appropriate to assume the objective of ensuring “universal health coverage” [13]. Furthermore, it is reinforced that the SUS is a public system financed by general taxes and with universal access. More than 70% of the Brazilian population depends exclusively on the SUS to access health care.

However, as already mentioned in this work, health education in the context of planetary health requires a paradigm shift involving the whole of society, empowering the population that needs to build its knowledge and increase its autonomy in individual and collective care. Moreover, health professionals and managers who support these professionals are also key players in this scenario. Thus, the incorporation of the concepts of planetary health into people's thinking, managers, legislators, and governors' practices is a very complex process, especially in a country with deep inequalities such as Brazil.

The health of communities and people is intrinsically related to planetary health. In the case of Brazil, this relationship becomes even more critical. It is a country with continental dimensions, a large population, a significant economy, and extraordinary natural resources, but with profound social inequalities. Brazil has a current population of 213,861,333 inhabitants, a GDP in 2020 of BRL 7.4 trillion, and an annual GDP per capita in 2019 of BRL 35,161.70 (∼ U$D 8800). Regarding the Brazilian health system, in 1980, the country had 0.98 doctors per 1000 inhabitants, a proportion of 1.68 in 2010, rose to 2.00 in 2015, and is currently at 2.4. In 2020, Brazil had 478,010 doctors, 342 medical schools, and 35,622 new positions [14].^1^

The size of the country's health system means it needs to incorporate actions focusing on the determinants of the health-illness process, individually and collectively. This necessarily implies the education and awareness of communities and people to preserve the environment in which they live, to adopt health-promoting behaviours, and consequently promote health in all dimensions, including planetary health, since their determinants have common roots. Health is a complex social phenomenon that depends on the social structure and setting in which it is observed, according to the Commission on Social Determinants of Health of the World Health Organization [15]. Thus, the health-disease process depends on the interaction, in each context, of determinants that can be grouped under the tripod of sustainable development: economic, social, and environmental protection [[16], [17], [18], [19], [20], [21]].

In this connection, all human health and well-being dimensions are affected by global environmental changes [22]. Therefore, reorganising health systems to implement and cooperate with planetary health presupposes a paradigm shift that ranges from process efficiency, the acquisition of supplies, and the use of sustainable energy sources to waste reduction and people's education, both for users and service providers.

According to the São Paulo Declaration on Planetary Health [31], signed in October 2021 by more than 250 organisations from 47 countries and representing all sectors of society, a change in the focus of health care is essential to a guide fundamentally aimed at disease prevention and health promotion. Furthermore, it presupposes incorporating health perspectives and practices beyond the traditional Western methods, including the knowledge of indigenous peoples and integrative health practices. In addition, to focus on the social and environmental determinants of health, both individual and collective, it is necessary to incorporate different sectors, such as education, transport, security, employment, leisure, housing and sanitation, and universal access to health services. Finally, it includes providing green spaces, guaranteeing the air, soil and water quality, and access to nutritious and healthy food, with particular attention to communities with greater social vulnerability [22].

In this scenario, Brazil has the world's fifth largest population and seventh largest economy [23,24], provided for in its Federal Constitution, the *Sistema Único de Saúde* (SUS, Unified Health System). It is based on structuring and organisational principles [25]^2^ that, in essence, incorporate principles that strengthen planetary health by recognising that health levels express the social and economic organization of the country, having as determinants and conditioning factors, among others: food, housing, basic sanitation, the environment, work, income, education, physical activity, transport, leisure and access to essential goods and services [26].^3^

The structuring principles of SUS refer to Universalization, Equity and Integrality. Universalisation presupposes health as a right of citizenship for all, and the Brazilian State must ensure that right. Equity aims to reduce inequalities with more excellent investment where shortages and needs are significant. On the other hand, Integrality considers people, integrating actions for health promotion, disease prevention and treatment and rehabilitation [25].^3^ In connection with the average health spending/year per capita in Brazil, the Federal Council of Medicine reports it was R$ 1398.53 (about U$D 350) by the three levels of government in 2019. The information considers the expenses of Public Health Actions and Services declared in the Information System on Public Health Budgets (SIOPS) of the Ministry of Health.

By law, each federative entity must invest a minimum portion of the tax resources and the constitutional and legal transfers. In the case of the States and the Federal District, this rate must be at least 12% of their total budgets, while in the case of municipalities, the base value corresponds to 15%. For the Federal Government, the amount is calculated: expenses paid plus remaining expenses payable from the previous year, restated for inflation. In 2019, expenditures at the three management levels reached R$292.5 billion. This amount includes the coverage of actions and services to improve the Unified Health System (SUS), such as the cost of the service network and employees' payments [27].^4^ Table 1 shows the per capita gross domestic product, the Union's public expenditure on health per capita and the number of physicians per 1000 inhabitants in the Brazilian States to detail health expenditures in Brazil [[28], [29], [30]]^4^.

**Table 1:** Gross domestic product per capita, public health spending by the Union, and the number of physicians per 1000 inhabitants in the Brazilian States.

States	GNP per capita (2019) (R$)^a^	Union spending on health per capita(2020)(R$)	Doctors/1000 inhab. (2020)
**North Region**			
Acre	17.722,41	476,64	1,20
Amapá	20.688,21	439,13	1,19
Amazonas	26.101,72	298,81	1,30
Pará	20.734,60	292,58	1,07
Rondônia	26.497,12	378,58	1,78
Roraima	23.593,84	429,34	1,61
Tocantins	25.021,80	512,58	2,01

**Northeast Region**			
Alagoas	17.667,79	440,43	1,58
Bahia	19.716,21	375,35	1,64
Ceará	17.912,17	399,03	1,65
Maranhão	13.757,94	347,99	1,08
Paraíba	16.919,84	431,63	2,04
Pernambuco	20.702,30	405,05	2,02
Piauí	16.125,00	467,68	1,60
Rio Grande do Norte	20.342,11	400,92	1,92
Sergipe	19.441,23	426,41	1,90

**Midwest Region**			
Distrito Federal	90.742,75	1.973,66	5,11
Goiás	29.732,40	340,65	2,28
Mato Grosso	40.787,32	352,47	1,91
Mato Grosso do Sul	38.482,83	405,67	2,36

**Southeast Region**			
Espírito Santo	34.177,05	334,18	2,75
Minas Gerais	30.794,04	386,96	2,66
Rio de Janeiro	45.174,08	464,31	3,70
São Paulo	51.140,82	308,15	3,20

**South Region**			
Paraná	40.788,77	380,41	2,49
Rio Grande do Sul	42.406,09	499,11	2,89
Santa Catarina	45.118,41	372,30	2,64
**Brazil**	**35.161,70**	**703,55**	**2,27**

a (R$- Brazilian currency; 1 R$ is equivalent to 0.19 US$ on October 28, 2022).

The guarantee of a robust health system accessible to all, equitable, based on health promotion and the prevention of injuries and diseases and the preservation of the environment, to guarantee access to the basic needs of human beings, is essential for a change of focus towards collective and planetary health. In this connection, Whitmee et al. [1] claim that health systems, and their professionals, have been acting strategically in the face of the challenge of integrating policies that promote the reduction of inequalities and environmental impacts, which, however, encourage greater resilience to environmental changes and health and environmental sustainability. These movements have been occurring in all sectors and favour planetary health within health systems and populations.

## 3 Trends in health education

In this scenario of planetary health, health education can collaborate to preserve and promote health in all dimensions. The World Health Organization fostered knowledge about the relationship between health and social determinants and the health-disease process through the Commission on Social Determinants of Health. Health is framed within the social justice model, a complex social phenomenon [15].

Health education represents a field of disputes between social projects and worldviews that are updated in conceiving and organising discourses and practices related to education in health. Therefore, understanding the underlying conceptions of education, health, and society is necessary. The Ministry of Health in Brazil defines health education as an educational process for the construction of knowledge in health aimed at the thematic appropriation by the population, constituting a set of sector practices that contribute to increasing the autonomy of people in their care and to the discussions with professionals and managers to achieve health care according to their needs. Health education practices involve three fundamental elements: health professionals who value disease prevention and health promotion as much as curative practices; managers who support these professionals; and the population that needs to build its knowledge and increase its autonomy in individual and collective care. Although the definition of the Ministry of Health presents elements that presuppose this interaction for the development of this process, there still needs to be more consistency between theory and what happens in practice [31]. In Brazil, two approaches antagonise traditional and popular health education.

The traditional hegemonic model centralises power in health professionals, who are holders of the necessary knowledge to lead a healthy life, known as the biomedical model. Such an approach does not privilege the subject's autonomy, as it prescribes education vertically [32]. This education model presupposes the adoption of healthy habits and behaviours through the persuasion of individuals, such as giving up smoking, getting vaccinated, using hygiene practices, and taking preventive exams. It occurs through contact with mass communication vehicles or access to information provided by the educator. Formal health education is tackled mainly by the Brazilian Ministry of Education. The Ministry of Health attaches little attention to it, mostly limited to non-formal and ad hoc approaches. Health education, from this perspective, promotes conscious decision-making by the population, who are informed about the risks of certain behaviours and entirely responsible for their health condition; that is, a practice that reveals the functioning of public services and governments uncommitted to the development of autonomy and social empowerment [32].

Contrary to traditional education, the other aspect has been called popular health education and seeks autonomy, people's participation, and dialogue between knowledge and practices [32]. This approach pedagogically addresses the process of popular participation, stimulating collective forms of learning and investigation, promoting the growth of critical analysis skills on reality, and improving strategies for facing and coping with life situations that determine the health-disease process [50]. It is a strategy for building popular participation in redirecting social life and reinforces the SUS organisational principle of Popular Participation. Its method assumes that popular classes have their dynamics of recognition and confrontation with the health-disease process and its healing processes acquired in everyday life. This knowledge should be acknowledged and incorporated into health practices [32]. A horizontal relationship is developed between health professionals, considered mediators, and the community in an educational dialogue accompanied by movements for community strengthening, seeking to create fairer social relationships. Such an approach seems to be more in line with what the São Paulo Declaration on Planetary Health advocates [6] when it points out that it is essential to change the healthcare focus, which presupposes incorporating health perspectives and practices beyond traditional methods, as well as the inclusion of integrative health practices. This necessarily implies critical awareness of individuals and communities in an empowered society.

There is promising theoretical and methodological development in health education in Brazil. However, such reflections have yet to be consistently translated into service practice, causing a gap between theory and practice [33]. Thus, popular education is mainly carried out as ideology, by health workers who believe in their transforming power, not only for individuals' lives but also for society's general organization. However, the practice of health education in Brazil continues to be, for the most part, the traditional prescriptive, which contributes only marginally to the necessary critical education aimed at collective and planetary health.

## 4 Methodology

**Fig. 1:**
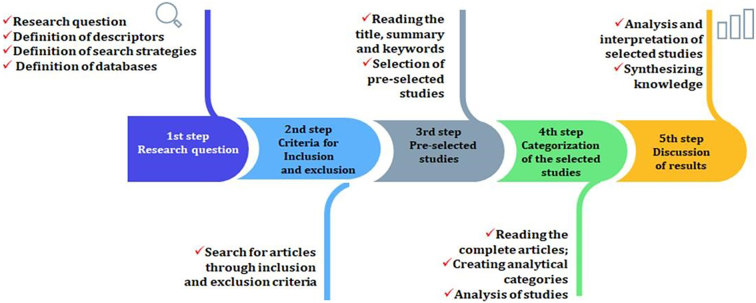
The steps of the literature review.

This study involved exploratory research with a qualitative approach based on a literature review and quantitative data on the Brazilian health situation. For the literature review, the integrative review technique was used, which contributed to the composition of 1, 2 (Introduction; Theoretical framework) and founded the “Discussion” section, Scopus, Web of Science, Science Direct, PubMed and Scielo databases were used, with the following search strategies: “planetary health” AND Brazil; “planetary health” AND “Latin America”; “planetary health” AND Anthropocene; “planetary health” AND “climate change”; “planetary health” AND SDGs; “planetary health” AND “human health”. The literature review steps followed Fig. 1, in English and Portuguese, without time limitation.

After following the steps in Fig. 1, it was possible to reach a portfolio of 59 scientific articles which met the eligibility criteria (inclusion and exclusion criteria). The studies of the 57 articles were essential to systematise knowledge, which is presented in the results section. Furthermore, due to its importance concerning the topic under study, this set of papers was also fundamental to support the discussion of the present study. To complement, it was also necessary to study essential documents belonging to different national and international institutions: World Health Organization, Pan American Health Organization, Osvaldo Cruz Foundation / Brazil, Brazilian legislation, and, mainly, documents from the Unified Health Service (SUS).

## 5 Presentation and discussion of the results

Planetary health can be understood as a great ally to different economic sectors, including energy, agriculture, water, fisheries, and health, in the struggle to change the tax and subsidy scenario at the global and national levels. However, on the other hand, planetary health is hostage to the need to transform the economy [1]. Therefore, it appears that the growing depletion and scarcity of the Earth's natural resources have represented a threat, not only to planetary health but mainly to human health, due to its dependence on the health of the planet (The Convention of the United Nations Framework on Climate Change - UNFCCC, 2020), marked by a high risk of deterioration and exposure to risk [54].

Therefore, several sources [34,35] State that the beginning of the 21st century has brought new questions to human society that involve the urban situation under the horizon of planetary health. Currently, planetary health has incorporated into its conceptual proposal the notion of inseparability and approximation to social issues, such as structural racism and poverty, which have been intensified by the inequalities resulting from the pandemic and have been represented by protests such as Black Lives Matter, as well as the occurrence of weather attacks [36,37].

The quest to safeguard planetary and human health has taken this mission to protect cities and urban governance, even though health and equity are not necessarily prioritised in decision-making processes [38]. Furthermore, according to Schwerdtle et al. [39], when it comes to the biomedical approach to health, we can emphasise that it works in a complementary way to planetary health, based on social and environmental models, in addition to requirements such as systemic and preventive thinking and public health. Therefore, it is noted that the concept of planetary health falls within the scope of knowledge about planetary functioning and the health of the Earth and its inhabitants through the criteria of integration and interconnectivity [40]. Society in all sectors, communities, all levels of government, and educators believe and focus actions on health promotion measures. Adopting health-promoting behaviours by people supported by public policies that facilitate healthy behaviours in all sectors of society can positively impact planetary health, as the exact causes determine both people's and the planet's health.

Given the gap between environmental sustainability and public health, this challenge, in line with these transdisciplinary approaches, has also been supported by the capacity of planetary health to establish a common language [41,42]. On the other hand, from the point of view of economic growth, Egger et al. [43] and Lowe [44] observe the complexity load of the effects generated when this criterion interacts with human health and planetary health. However, it is also essential to add that the fundamentals that govern planetary health also influence the context of education for sustainable health, and this enhances the systems - human and terrestrial, biological or social - that represent this interdependence of life, which is characteristic of planetary health [45]. It is important to reinforce that education for sustainable health implies a high level of education for people, including health education supported by public policies that make healthy choices more accessible for people and different sectors of society.

It is well known that reducing environmental impacts and caring for the environment directly impact aspects such as improving and maintaining human health. In other words, integrating conditions related to human health and the ecological systems that make up nature form the concept of planetary health [1,22,46]. As argued by authors [47], the principles of planetary health are rooted in the beings that inhabit the planet, based on the existence of an interconnection process, and go further, as it carries traditional indigenous knowledge being defended by these populations. The role played by planetary health refers, therefore, to the application of knowledge in the world, about the species and environments that form it [48]. In this sense, the author [49] also mentions that planetary health has recently benefited from technological systems, such as artificial intelligence and digital health.

Planetary health is known to advocate systems thinking [[50], [51], [52]]. Thus, authors [51] complement authors [52], providing evidence that environmental and human health, subject to solid interactions management, reflect the contributions of integrated and transdisciplinary research aimed at this scenario. Therefore, sustainability indicators can become strategic variables in this decision-making universe, considering sustainable performance management formed by a set of planetary health indicators [52]. In the context of food production, a dietary pattern of health that meets planetary health must be represented by planetary biophysical limits, surpassing criteria of nutritional needs, and being used in the assessment of sustainability [[53], [54], [55], [56]]. Thus, the relationship between the posture adopted by human beings in the face of natural systems and their consequences on public health constitutes concerns inherent to planetary health [57,58].

Given the context of the values preached by planetary health, this distance itself from materialism but presents a positive correlation with elements such as empathy, pro-environmental attitudes and humanitarianism [44,59,60]. In addition, to transforming agents, one can mention the role of cities in the face of planetary health as centres responsible for encouraging, or not, the realisation of environmental changes, of a global nature, given the highly vulnerable profile and the leadership role assumed by cities [[61], [62], [63]].

Finally, in connection with the values advocated by planetary health, they are distant from materialism but show a positive correlation with elements such as empathy, pro-environmental attitudes, and humanitarianism [[64], [65], [66]]. Finally, about transforming agents, the role of cities in terms of planetary health should be mentioned, as they are centres responsible for encouraging, or not, the performance of environmental changes of a global nature, given the highly vulnerable profile and the leading role assumed by cities [[61], [62], [63]].

## 6 Conclusions

This article addresses health education in Brazil and its relationship with planetary health, making notes on the Global Sustainable Development Goals/SDGs. Planetary health in Brazil, considering the last decades, faces some challenges to be overcome, such as socioeconomic inequalities among its people; the risks to which Amazonian biodiversity and its ecosystem service of climate regulation are subjected; the increase in deforestation, forest fires, greenhouse gas emissions, and air pollution, with a significant impact on climate change. These challenges are fundamental to be studied and resolved, as they impact the health of people and the Brazilian ecosystem locally and globally on the health of planet Earth.

Brazil has the Unified Health System/SUS that has brought significant improvements in Brazilians' health in recent decades, based on Universalisation, Equity, and Integrality. The SUS incorporated determining and conditioning factors into its practices, such as care for food, housing, sanitation, the environment, and access to essential goods and services, in line with the principles of planetary health. In addition, the number of doctors increased from 0.98 to 2.4 per 1000 inhabitants from 1980 to 2021. These improvements were only possible through investments in the training of health professionals, with an interdisciplinary vision, with the support of other professionals, mainly those related to the environment.

As all dimensions of health and human well-being are affected by global environmental changes, the reorganisation of health systems considering the planetary health approach needs a paradigm shift, with the insertion of process efficiency practices, use of sustainable energy sources for waste reduction, and education for both users and service providers. The São Paulo Declaration on Planetary Health (October 2021) was a turning point towards a new approach to health. The declaration focused, in addition to universal access to health services, on other fundamental aspects: the incorporation or provision of disease prevention and health promotion; indigenous knowledge and integrative health practices; the education, transport, security, employment, leisure, housing and sanitation sectors; the green spaces that guarantee environmental quality; access to nutritious and healthy food, especially in socially vulnerable communities. To carry out the actions provided for in the declaration, it is necessary to constantly invest in health education and health promotion policies, understanding that all health actions can generate an environmental impact and reduce inequalities (social determinants of health) and environmental impacts. This way, environmental health, and sustainability can promote resilience to environmental changes.

Education and health are social areas that influence each other. However, in Brazil, two approaches antagonise health education I) traditional hegemonic (vertical relationship Professionals-Communities) and II) popular health (horizontal relationship Professionals-Communities) education models. The former centralises health professionals' power to promote conscious decision-making by an informed population without privileging the subject's autonomy and social empowerment. The latter seeks autonomy, people's participation, and dialogue between knowledge and practices, reinforcing the SUS organisational principle of Popular Participation. Furthermore, the horizontal participatory approach fits the São Paulo Declaration on Planetary Health regarding the need to change the healthcare focus, with perspectives and practices beyond traditional methods and integrative health practices, leading to social empowerment.

This paper has some limitations. The first one is the fact that is used a qualitative approach to the literature review. This has limited the scope of the papers analyzed. Secondly, the focus on quantitative data on Brazilian health has relied on official sources. Finally, it does not entail the latest data (e.g. for the year 2021) since these still need to be made available. However, despite these constraints, the paper is a welcome addition to the literature in that it outlines the potential and constraints concerning planetary health in Brazil.

Some measures which should be considered as part of efforts to realise the potential of planetary health are:

a) A national strategy on planetary health which goes across the ministries of health and environment and reaches synergies with the budgetary provisions of both ministries, hence absorbing any additional costs
b) The introduction of the principles of planetary health as part of the country's national health policies
c) The inclusion of matters related to planetary health as part of the training of health professional
d) Due to consideration of planetary health as part of the training of medical students

This list is incomplete and exemplifies what can be done. There should be more practical developments in Brazil's health education and service practice. Horizontal popular education remains mostly an ideology sustained by health workers, while traditional prescriptive education prevails. Therefore, although positive improvements towards achieving collective and planetary health that advocates for empathy, pro-environmental attitudes, and humanitarianism occurred over the last few decades, implementing the necessary horizontal education approach still needs to be improved.

## Author contributions

WLF: designed data collection tools; monitored data collection for the whole study, wrote the analysis plan, drafted and revised the paper, coordinated the paper. JS: designed data collection tools; monitored data collection for the whole study, wrote the analysis plan, drafted the paper. AA: designed data collection tools; monitored data collection for the whole study, drafted the paper. MM: designed data collection tools; monitored data collection for the whole study, drafted the paper. JT: designed data collection tools; monitored data collection for the whole study, wrote the analysis plan, revised the paper. GN: cleaned and analyzed the data; drafted and revised the paper. All authors were responsible for ensuring the accuracy and integrity of their contributions and approved the final manuscript.

## Funding

This paper was funded by the International Climate Change Information and Research Programme (ICCIRP), of the Hamburg University of Applied Sciences (HAW), Hamburg, Germany.

## Declaration of Competing Interest

The authors declare that the research was conducted in the absence of any commercial or financial relationships that could be construed as a potential conflict of interest.

## Data Availability

Data will be made available on request.

## Footnotes

1 https://www.ibge.gov.br/apps/populacao/projecao/index.html.

2 https://www.gov.br/saude/pt-br/assuntos/saude-de-a-a-z/s/sistema-unico-de-saude-sus-estrutura-principios-e-como-funciona

3 http://www.planalto.gov.br/ccivil_03/leis/l8080.htm

4 https://portal.cfm.org.br/noticias/brasil-gasta-r-383-ao-dia-com-a-saude-de-cada-habitante-2/
